# Risk of Severe Acute Respiratory Syndrome Coronavirus 2 Infection Following Prior Infection or Vaccination

**DOI:** 10.1093/infdis/jiae130

**Published:** 2024-05-08

**Authors:** Joseph E Ebinger, Nancy Sun, Sandy Y Joung, John Michael S Sanchez, Minhao Wang, Yunxian Liu, John C Prostko, Edwin C Frias, James L Stewart, Mallory Heath, Brian L Claggett, Susan Cheng, Kimia Sobhani

**Affiliations:** Department of Cardiology, Smidt Heart Institute, Cedars-Sinai Medical Center, Los Angeles, California; Department of Cardiology, Smidt Heart Institute, Cedars-Sinai Medical Center, Los Angeles, California; Department of Cardiology, Smidt Heart Institute, Cedars-Sinai Medical Center, Los Angeles, California; Department of Cardiology, Smidt Heart Institute, Cedars-Sinai Medical Center, Los Angeles, California; Department of Cardiology, Smidt Heart Institute, Cedars-Sinai Medical Center, Los Angeles, California; Department of Cardiology, Smidt Heart Institute, Cedars-Sinai Medical Center, Los Angeles, California; Applied Research and Technology, Abbott Diagnostics, Abbott Park, Illinois; Applied Research and Technology, Abbott Diagnostics, Abbott Park, Illinois; Applied Research and Technology, Abbott Diagnostics, Abbott Park, Illinois; Department of Cardiology, Smidt Heart Institute, Cedars-Sinai Medical Center, Los Angeles, California; Cardiovascular Division, Brigham and Women’s Hospital, Boston, Massachusetts; Department of Cardiology, Smidt Heart Institute, Cedars-Sinai Medical Center, Los Angeles, California; Department of Pathology and Laboratory Medicine, Cedars-Sinai Medical Center, Los Angeles, California

**Keywords:** COVID-19, SARS-CoV-2, vaccination, immunity, aging

## Abstract

**Background:**

The extent to which infection versus vaccination has conferred similarly durable severe acute respiratory syndrome coronavirus 2 (SARS-CoV-2) immunity during the Omicron era remains unclear.

**Methods:**

In a cohort of 4496 adults under continued serological surveillance throughout the first year of Omicron-predominant SARS-CoV-2 transmission, we examined incidence of new infection among individuals whose last known antigenic exposure was either recent (<90 days) or remote (≥90 days) infection or vaccination.

**Results:**

We adjudicated 2053 new-onset infections occurring between 15 December 2021 through 22 December 2022. In multivariable-adjusted analyses, compared to individuals whose last known exposure was remote vaccination, those with recent vaccination (odds ratio [OR], 0.82 [95% confidence interval {CI}, .73–.93]; *P* = .002) or recent infection (OR, 0.14 [95% CI, .05–.45]; *P* = .001) had lower risk for new infection within the subsequent 90-day period. Given a significant age interaction (*P* = .004), we found that remote infection compared to remote vaccination was associated with significantly greater new infection risk in persons aged ≥60 years (OR, 1.88 [95% CI, 1.13–3.14]; *P* = .015) with no difference seen in those <60 years (1.03 [95% CI, .69–1.53]; *P* = .88).

**Conclusions:**

During the initial year of Omicron, prior infection and vaccination both offered protection against new infection. However, remote prior infection was less protective than remote vaccination for individuals aged ≥60 years. In older adults, immunity gained from vaccination appeared more durable than immunity gained from infection.

## Teaser

In a cohort study of 4496 community-dwelling adults during the initial year of Omicron, prior infection and vaccination both offered protection against new SARS-CoV-2 infection. Importantly, remote prior infection was less protective than remote vaccination for individuals aged ≥60 years.

Now that public health efforts have transitioned from pandemic to endemic management of severe acute respiratory syndrome coronavirus 2 (SARS-CoV-2) risks, attention has focused on the population immunity afforded by prior infections and vaccinations. In particular, the US Food and Drug Administration and other governmental agencies have deliberated the possibility that SARS-CoV-2 could be considered similar to respiratory viruses such as influenza and respiratory syncytial virus [1]. If current and future forms of SARS-CoV-2 demonstrate seasonal patterns in transmission and associated outcomes that are indeed similar to those of other respiratory pathogens, then a reasonable public health strategy could involve offering updated vaccinations on an annual or seasonal basis for all or a subset of the populations at risk [2]. The challenge to formulating public health guidance is the repeatedly cited dearth of contemporary data regarding the degree and duration of immunity offered by prior infection compared to prior vaccination [3]. We and others have reported previously on how both types of prior SARS-CoV-2 exposure, infection or vaccination, provide augmented immunity but for a limited time period that varies based on individual-level and viral variant–related factors [4–6]. The extent to which prior findings have persisted through the era of SARS-CoV-2 transmission dominated by Omicron subvariants remains unclear. Therefore, we conducted a longitudinal study involving clinical and serological surveillance to evaluate the relative immunologic protection afforded by either prior infection or prior vaccination against any new-onset infection occurring during the Omicron era.

## METHODS

### Study Cohort

We enrolled into a longitudinal SARS-CoV-2 surveillance study a total of 9312 individuals who were registered as employees or patients at Cedars-Sinai Medical Center, an academic medical center that serves the diverse urban metropolis of Los Angeles County [5]. All enrolled study participants were asked to complete baseline and serial surveys on medical comorbidities, SARS-CoV-2 exposures (including the number and timing of infections and vaccination doses), and symptoms, according to previously described protocols [7]. Self-reported medical comorbidities were verified using medical records and used to calculate a comorbidity index using the validated Elixhauser method [8]. All participants were invited to contribute up to monthly blood draws for serial SARS-CoV-2 antibody assays, described below, as part of ongoing serological surveillance. The current study was designed to investigate the extent to which a prior SARS-CoV-2 antigenic exposure (infection or vaccine) conferred immunity to a new-onset infection during the Omicron era, which began on 15 December 2021 in our geographic region [3, 7]. Thus, for the current analysis, we included 4557 participants who experienced at least 1 prior SARS-CoV-2 exposure, either infection or vaccination, prior to 15 December 2021 and provided a minimum of 2 blood samples for serology, at least 1 of which was drawn after 15 December 2021. We excluded 44 participants without follow-up survey data after 1 December 2021 and 17 individuals aged <18 years or with data missing on key covariates, leaving 4496 participants eligible for analyses (Supplementary Figure 1). All study participants provided written informed consent for all protocols, which were approved by the Cedars-Sinai institutional review board. This report follows the Strengthening the Reporting of Observational Studies in Epidemiology (STROBE) reporting guideline for observational studies.

### Clinical and Prior SARS-CoV-2 Exposure Assessments

Presence or absence of comorbidities was ascertained using self-reported data collected via periodic surveys and stored in our REDCap electronic survey database, then verified using medical history data extracted by algorithm-based scripts from the electronic health record (EHR); our institutional EHR includes robust data on all patients cared for across the Cedars-Sinai Delivery Network, though not directly linked outside regional or national repositories. Historical infection events data were ascertained based on documentation in the EHR, self-report verified using outside records of polymerase chain reaction (PCR) or other testing, and evidence of prior increase in nucleocapsid immunoglobulin G (IgG-N) antibody level to ≥1.4 index [5, 9]. Historical vaccination events were ascertained based on vaccination type, dosing, and date of administration information collected from the EHR and self-report verified by review of vaccination card or outside records. All staff involved in manual verification protocols used standardized data capture instruments that included explicitly defined variable fields, and all verification procedures were performed in batch fashion for the source cohort study as a whole and, thus, all staff were blinded to the study hypothesis for this analysis. All historical infection and vaccine events occurring prior to the start of the Omicron era surveillance period (ie, beginning 15 December 2021) were categorized as recent in timing (<90 days) or remote in timing (≥90 days); this cutoff period of 90 days was selected based on the US Centers for Disease Control and Prevention’s recommended length of time to wait between a SARS-CoV-2 infection and receiving a vaccination.

### New-Onset SARS-CoV-2 Infection Assessment and Serological Assays

New-onset SARS-CoV-2 infection events were ascertained using data collected through the following protocols: documentation in the EHR, self-report verified using outside records, and substantial increase in anti–IgG-N antibody level (as described below) given the ability of this serological measure to indicate asymptomatic as well as symptomatic infection events despite previous or recent vaccinations [7]. The EHR medical records of enrolled participants were screened for positive SARS-CoV-2 PCR results and *International Classification of Diseases, Tenth Revision* (*ICD-10*) code documentation of coronavirus disease 2019 (COVID-19). The date of the positive test or code was considered the date of infection. For patients who self-reported a COVID-19 infection and from whom temporally correlated PCR or *ICD-10* documentation was not present in the EHR, medical charts were manually reviewed for documentation of infection in provider notes; only self-reported events confirmed by medical records were considered true infections. Finally, an IgG-N index level rise to ≥1.4 on serology testing was considered indicative of an infection event having occurred. All serial serological measures were performed using Abbott Architect immunoassays (Abbott Park, Illinois) to quantify both circulating levels of SARS-CoV-2 anti–spike protein IgG (IgG-S) and IgG-N antibodies, as reported previously [4, 9–11]. Ascertainment of distinct new infection events were determined according to the algorithm outlined in Supplementary Figure 2.

### Statistical Analyses

**Figure 1.**
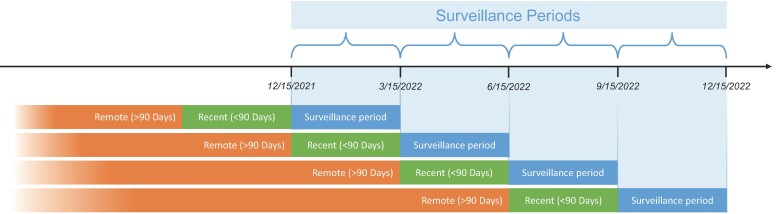
Study design overview. Over the course of the study period, which commenced at the start of the Omicron era in our region, participants were under surveillance for new infection during consecutive 90-day surveillance periods. For each participant, exposure group status (remote or recent exposure, infection or vaccination type of exposure) was updated prior to the beginning of each surveillance period.

We categorized participants by “exposure group” defined based on last known SARS-CoV-2 exposure: recent infection (<90 days prior), recent vaccination (<90 days prior), remote infection (≥90 days prior), or remote vaccination (≥90 days prior). In multivariable-adjusted analyses, we accounted for time-dependent factors by considering prior exposures and new infection events during sequential 90-day time periods (starting from 15 December 2021) to approximate the length of a typical surge. We also selected surveillance periods of 90 days to minimize the effect of infection risk changing over time, as shown in Supplementary Figure 3. Notably, exposure group status was time-updated for each participant whenever there occurred a new infection or vaccination event (Figure 1). For example, if a participant originally in the remote infection group then subsequently received a vaccine in the 90 days following 15 December 2021, for analyses of infection risk during the next time period, their status was updated as being in the recent vaccination group. In addition to exposure group status, all covariates were also updated at the start of each 90-day time period and any participant who developed a new infection event was then removed from all subsequent time period analyses. We used multivariable-adjusted logistic regression with clustered standard errors, accounting for repeated measures among participants, to estimate odds of new infection within each sequential 90-day time period, where the predictors of primary interest were type and timing of last known exposure. We iteratively estimated odds of new infection in reference to each of the different exposure groups so that results could be interpreted from the perspectives of individuals with either remote or recent exposures; however, we prioritized presenting results in reference to remote exposures given that the remote exposure scenario represents the most prevalent real-world situation for the majority of individuals in the population. The primary model adjusted for characteristics previously reported in association with measures of SARS-CoV-2 immunity [5] including age, sex, race, ethnicity, healthcare employee status, Elixhauser comorbidity index, and total number of prior exposures in addition to time window (ie, sequential 90-day time periods starting from 15 December 2021, during which background infections rates and predominant subvariant types tended to shift). We secondarily used multiplicative interactions terms to assess for effect modification by demographic, clinical, and exposure variables. Given previously reported age-based differences in immune susceptibility to infection, we prioritized tests of interaction by age using a Wald test with 3 degrees of freedom. In secondary analyses, we repeated analyses using parsimonious models that included only covariates that were significantly associated with the outcome in the primary analyses. We conducted all statistical analyses using R software (version 4.2.1) and considered a 2-tailed *P* <.05 statistically significant.

## RESULTS

For the total study cohort of 4496 participants at baseline, the mean age was 54 (standard deviation, 15) years with 37% males and 36% self-identified as non-White (Table 1). Characteristics of the study cohort were relatively comparable to those of the original source cohort and a random sampling of the larger health system cohort of patients, although study sample participants tended to be younger and were more frequently females and of Asian race (Supplementary Table 1). For study cohort, across sequential surveillance time periods, age at risk for new infection increased along with aggregate comorbidity burden represented by the Elixhauser index and prevalence of select comorbidities (including hypertension, heart disease, cancer, and organ transplant status) while prevalence of healthcare employee status decreased (Supplementary Table 2).

**Table 1.** Study Cohort Characteristics

Characteristic	Total Cohort (N = 4496)
Age, y, mean (SD)	53.6 (14.8)
Male sex	1652 (36.7)
Hispanic ethnicity	562 (12.5)
Race	
American Indian or Alaska Native	15 (0.3)
Asian	680 (15.1)
Black or African American	208 (4.6)
Native Hawaiian or other Pacific Islander	53 (1.2)
White	2881 (64.1)
Other	206 (4.6)
Multiple	175 (3.9)
Unknown	278 (6.2)
Healthcare employee	1897 (42.2)
Past medical history	
Hypertension	1202 (26.7)
Diabetes	426 (9.5)
Coronary heart disease or heart failure	789 (17.5)
Asthma or COPD	689 (15.3)
Cancer	685 (15.2)
Autoimmune disease	607 (13.5)
Organ transplant recipient	548 (12.2)
Elixhauser comorbidity index, mean (SD)	4.50 (11.16)

Data are presented as No. (%) unless otherwise indicated.

Abbreviations: COPD, chronic obstructive pulmonary disease; SD, standard deviation.

### New-Onset Infections During the Omicron Era

During the study surveillance period, a total of 2053 participants developed new infections with a temporal frequency that mirrored temporal patterns of infection risk indicators that were observed in the source cohort as well as in our local region, on the background of shifting predominance of Omicron subvariants over time (Supplementary Figures 4 and 5).

### Multivariable-Adjusted Risks for New Infections During the Omicron Era

In multivariable-adjusted analyses of infection risk in relation to last known SARS-CoV-2 exposure, which was time-updated through the surveillance period, we found that remote compared to recent exposures were consistently associated with greater risks for new infection (Table 2) after accounting for the demographic, clinical, and exposure factors listed in Supplementary Tables 3 and 4. In particular, compared to individuals with remote exposures (Table 2), the risk for new infection was significantly lower for those whose last known exposure was recent infection (odds ratio [OR], 0.14 [95% CI, .05–.45] for recent infection vs remote vaccine and OR, 0.11 [95% CI, .03–.36] for recent infection vs remote infection) or vaccine (OR, 0.82 [95% CI, .73–.93] for recent vaccine vs remote vaccine and OR, 0.64 [95% CI, .46–.89] for recent vaccine vs remote infection). Notably, for individuals whose last known exposure was remote vaccination (Table 2), the risk magnitude of new infection risk was greater for those whose last known exposure was remote infection (OR, 1.29 [95% CI, .94–1.76]), although this difference was not statistically significant in the overall cohort (*P* = .12). In sensitivity analyses using 45-day instead of 90-day periods of surveillance for new infection, results were similar (Supplementary Table 5). Results were also similar for parsimonious model analyses (data not shown).

**Table 2.** Multivariable-Adjusted Risks for New Infection in Relation to Remote Exposures During the First Year of Omicron

Referent	Exposure Group	Odds Ratio (95% CI)	*P* Value
Remote vaccine	Recent vaccination	0.82 (.73–.93)	.**002**
	Remote infection	1.29 (.94–1.76)	.12
	Recent infection	0.14 (.05–.45)	.**001**
Remote infection	Remote vaccination	0.78 (.57–1.06)	.12
	Recent vaccination	0.64 (.46–.89)	.**007**
	Recent infection	0.11 (.03–.36)	**<**.**001**

Estimates and corresponding *P* values (with statistical significance denoted in bold) are shown for odds of new-onset infection within the subsequent 90-day period, where the primary covariates of interest are type and timing of last known exposure. The last known exposure was updated over time to reflect whether the last known exposure was recent within 90 days or remotely occurring 90 days or more previously. All estimates are derived from the multivariable model that adjusted for age, sex, race, ethnicity, healthcare employee status, Elixhauser comorbidity index, and total number of prior exposures in addition to time window (ie, sequential 90-day time periods starting from 15 December 2021, during which background infection rates and predominant subvariant types tended to shift).

Abbreviation: CI, confidence interval.

### Age-Stratified Risks for New Infections During the Omicron Era

In analyses testing for effect modification by demographic and clinical covariates, a significant interaction with the exposure group variable was observed only for age (*P* = .004). Therefore, we performed age-stratified analyses and selected age 60 years as the threshold given the distribution of age and frequency of events in our cohort (Supplementary Table 1). Among individuals aged <60 years, recent compared to remote exposures and particularly recent infection were associated with significantly lower risk for new infection (Table 3 and Supplementary Table 6), as seen in the overall cohort. Among individuals aged ≥60 years, all exposure types were associated with significantly lower risk for new infection when compared to remote infection—including remote vaccination (Supplementary Table 6). Accordingly, when compared to remote vaccination (Table 3), remote infection was associated with significant 88% greater odds for new infection (*P* = .015). These findings remained consistent with and without adjustment for clinical comorbidity burden of represented by the Elixhauser comorbidity index. Sensitivity analyses using subsequent 45-day periods demonstrated similar results (Supplementary Table 7). Results were also similar for parsimonious model analyses (data not shown).

**Table 3.** Multivariable-Adjusted Risks for New Infection in Relation to Remote Exposures During the First Year of Omicron, by Age Group

Referent	Exposure Group	Age <60 Years		Age ≥60 Years	
		Odds Ratio (95% CI)	*P* Value	Odds Ratio (95% CI)	*P* Value
Remote vaccine	Recent vaccination	0.76 (.65–.89)	.**001**	0.92 (.75–1.14)	.44
	Remote infection	1.03 (.69–1.53)	.88	1.88 (1.13–3.14)	.**015**
	Recent infection	0.12 (.03–.49)	.**003**	0.22 (.03–1.63)	.14
Remote infection	Remote vaccination	0.97 (.65–1.45)	.88	0.53 (.32–.89)	.**015**
	Recent vaccination	0.74 (.49–1.11)	.15	0.49 (.28–.84)	.**01**
	Recent infection	0.12 (.03–.50)	.**004**	0.12 (.02–.92)	.**041**

Estimates and corresponding *P* values (with statistical significance denoted in bold) are shown for odds of new-onset infection within the subsequent 90-day period, where the primary covariates of interest are type and timing of last known exposure. The last known exposure was updated over time to reflect whether the last known exposure was recent within 90 days or remotely occurring 90 days or more previously. All estimates are derived from the multivariable model that adjusted for sex, race, ethnicity, healthcare employee status, Elixhauser comorbidity index, and total number of prior exposures in addition to time window (ie, sequential 90-day time periods starting from 15 December 2021, during which background infection rates and predominant subvariant types tended to shift).

Abbreviation: CI, confidence interval.

## DISCUSSION

In a diverse cohort of >4400 adults living in the greater Los Angeles County region, we observed that the degree and duration of SARS-CoV-2 immunity experienced during the first year of Omicron transmission varied by prior type and timing of prior antigenic exposure. Overall, we found that remote exposures (≥90 days) compared to recent exposures (<90 days) offered substantially less protection from new infection, as expected. Among persons with remote exposures, the protection gained from remote infection compared to remote vaccination was similar for individuals aged <60 years but was substantially reduced for individuals aged ≥60 years. Among persons with recent exposures, the magnitude and durability of protection was greater from infection than from vaccination but only for individuals aged <60 years. These results indicate that although immunity is gained from both infection and vaccination, certain individuals—particularly older adults—derive less immune protection from infection than from vaccination and this difference is pronounced in situations where the last known exposure was over 90 days previously.

Our findings expand from previously published population studies showing that recent compared to remote infection was more protective [12, 13] and that a higher compared to lower number of vaccination doses, including more recently acquired booster doses, conferred greater protection from infection during the Omicron era [12–14]. Prior studies have also found that remotely acquired vaccination had protective effects that persisted through the time periods dominated by newly emerged Omicron variants [14]. Our findings extend from prior work not only by confirming the durability of vaccination-induced immunization, particularly in older adults [13], but also by directly comparing the efficacy of infection versus vaccination as the last known exposure. Given the contemporary context of now endemic SARS-CoV-2 transmission, with most of the population having experienced a last known remote rather than recent exposure, we prioritized analyses of future infection risk in reference to the last known exposure being a remote vaccination. In this scenario, a hypothetical switch from remote vaccination into the more recent vaccination or recent infection group would be associated with significantly lower risk for subsequent new infection—with findings driven by persons aged <60 years. In the alternate scenario, where the last known exposure was remote infection, a hypothetical switch into the recent vaccination group or recent infection group would also be associated with significantly lower risk for subsequent new infection—with findings this time driven by persons aged ≥60 years. Notably, in older persons, being in the remote vaccination group was also associated with a 47% lower risk than being in the remote infection group—corresponding, in turn, to an 88% greater new infection risk seen for remote infection compared to remote vaccination. In effect, older adults whose last known exposure was remote vaccination were more protected than older adults with a remote prior infection.

Our age-dependent results are concordant with a wealth of prepandemic evidence demonstrating age-based differences in responses to vaccinations as well as endemic infections and, in turn, longstanding recommendations for age-specific vaccination strategies [15–17]. Mechanisms underlying impaired immune responses seen with aging are multifactorial [18, 19]. Importantly, age-related reduction in immunological responses to both infection and to vaccination are more pronounced in the setting of stressors [20, 21]. Thus, responses to infection versus vaccination could be impaired to a greater degree given the stressors that accompany infection. Indeed, multiple studies have found that after SARS-CoV-2 infection, in particular, T-cell lymphopenia and reduced antigen-specific B cells are prominently seen in older adults [22, 23]. In contrast to the exposure of infection, the exposure of vaccination provokes an immune response in older compared to younger adults that appears attenuated by some measures and not others; accordingly, the need for multiple frequent repeated vaccination doses (ie, more frequent than annual) in older adults appears yet uncertain given the results from our study as well as others [13]. Taken together, our findings in context suggest that response to natural SARS-CoV-2 infection wanes faster than response to vaccination in older adults—and these effects are likely specific to advancing age, given that our models accounted for total number of prior SARS-CoV-2 exposures and generated consistent results with and without adjustment for burden of comorbidities.

Our results also specifically evaluate cross-protection between pre-Omicron and Omicron antigenic exposure. The decision to specifically evaluate this cross-protection was deliberately made given the recognized ongoing viral mutations related to immunologic pressures placed on the virus. Given that these mutations continue to occur, it is expected that hosts will be regularly exposed to different variants than previously immunologically encountered. Notwithstanding differences in potential future viral mutations that may convey varying degrees of immunologic evasion and associated differential risk, our results are reflective of the reality of the need to assess infection risk in the face of ongoing changes to SARS-CoV-2.

Several limitations of our study merit consideration. Whereas the nature of antigenic exposure from infection evolved over the study period due to shifting Omicron subvariant predominance, the majority (88%) of all vaccinations were monovalent given that bivalent vaccines were not available until the fall of 2021. During potential future surges, periodic shifts in predominant subvariants are also likely to outpace the availability of updated vaccines. Our study did not have genotyping data available for each of the infection events to allow for direct ascertainment of SARS-CoV-2 variant type and, in turn, analyses of how dynamic changes in circulating variants may have contributed to infection risks over time. We were unable to capture all possible infection events, despite use of serial antibody assays to detect seroconversion with high sensitivity and specificity [10]. Seroconversion events could not be uniformly verified by concurrent or contemporary PCR or rapid antigen testing in every case, and new infections occurring close in timing with a prior infection may not have been detected based on the IgG-N level remaining elevated. Thus, for several reasons, the established criteria used to quantify infections in this analysis may have underestimated the true frequency. Notably, our analyses revealed apparently paradoxical finding of lower infection risk seen in relation to certain conventionally higher risk traits including comorbidities and characteristics that correlated with higher comorbidity index in our cohort (eg, older age, non-White race). Given that our study design included a sampling bias toward an engaged subset of the population willing to enroll in COVID-19 studies, risk-avoidant behaviors among persons with self-recognized greater morbidity risks may well have contributed to these paradoxically lower infection risks. Although all study participants are patients receiving care within our health system, it remains possible that some patients received some clinical care at medical centers that are outside of our network with medical records systems that are not integrated with our EHR; we made every effort to obtain outside records for adjudicating externally sourced relevant medical information whenever possible, yet we cannot completely rule out the possibility of incomplete data on clinical predicators or outcomes for at least some participants. Additionally, we categorized individuals based on only their most recent exposure given inability to completely capture history of all prior exposures occurring from the beginning of the pandemic. Thus, our analysis could not consider the history and pattern of prior SARS-CoV-2 exposures and the social, behavioral, and biological factors that may contribute to an individual being assigned to one exposure group versus another (eg, last known exposure being remote infection vs remote vaccination). Therefore, our study design could not completely account for potential effects of selection and other sources of bias and confounding and so the findings should be interpreted with caution [24]. Finally, our study was conducted in the context of an employee and patient study based at a single health system in Los Angeles County; further studies are needed to assess the generalizability of our results. Additional studies are also needed to evaluate risks in persons with known alterations in native and acquired immune responses, such as immunocompromised states. Notwithstanding the limitations, study strengths included the prospective design with standardized protocols for serial collection and adjudication of data on SARS-CoV-2 exposures including repeated serological assays enabling surveillance for infections that could otherwise go undetected due to minimal or no symptoms [7]. Our study sample was also large in size as well as diverse, including healthcare employees and community-dwelling patients, representing the range of adults at risk for SARS-CoV-2 infections.

While recognizing contextual factors and limitations, our study results may be helpful in guiding vaccination strategies being developed and updated as communities have transitioned from managing pandemic to endemic levels of risk. In particular, the finding that remote infection offers substantially less protection than either remote or recent vaccination in adults aged ≥60 years suggests that older individuals in this scenario may benefit from receiving a booster vaccination approximately 90 days after recovering from an infection or prior to the start of a new surge period. By contrast, the finding that remote infection appears to offer similar immunity to remote or recent vaccination in adults aged <60 years suggests that booster vaccinations may not be needed as frequently for the average person in this age group. Further studies are needed to validate and expand from our results as part of the ongoing effort to inform optimal strategies for mitigating risks across the population at large.

## Supplementary Data

Supplementary materials are available at *The Journal of Infectious Diseases* online (http://jid.oxfordjournals.org/). Supplementary materials consist of data provided by the author that are published to benefit the reader. The posted materials are not copyedited. The contents of all supplementary data are the sole responsibility of the authors. Questions or messages regarding errors should be addressed to the author.

## Supplementary Material

jiae130_Supplementary_Data

## Notes

***Acknowledgments*.** We are grateful to all of our patients and to the frontline healthcare workers in our health system who continue to be dedicated to delivering the highest quality care. We would like to especially thank the staff of the Institute for Research on Healthy Aging.

***Data sharing*.** Requests for de-identified data may be directed to the corresponding authors (S. C. and J. E. E.) and will be reviewed by the Office of Research Administration at Cedars-Sinai Medical Center prior to issuance of data sharing agreements, which are designed to ensure patient and participant confidentiality.

***Disclaimer.*** The funding sources had no role in the design and conduct of the study; collection, management, analysis, and interpretation of the data; preparation, review, or approval of the manuscript; or decision to submit the manuscript for publication.

***Financial support***. This work was supported in part by the Erika J. Glazer Family Foundation and Sapient Bioanalytics, LLC.
