# Supplementary material for: Risk of Severe Acute Respiratory Syndrome Coronavirus 2 Infection Following Prior Infection or Vaccination
Source: J Infect Dis. 2024 May 8;230(3):e584–90. doi: 10.1093/infdis/jiae130 (PMC11420800; doi:10.1093/infdis/jiae130)
Supplement: jiae130_Supplementary_Data [file jiae130_supplementary_data.docx]

**Risk of SARS-CoV-2 Infection Following Prior Infection or Vaccination**

**Supplementary Materials**

| **sTable 1.** Characteristics of the study cohort, source cohort, and health system cohort | 2 |
| --- | --- |
| **sTable 2.** Study cohort characteristics by time periods of eligibility for new infection | 3 |
| **sTable 3.** Risks for infection during the first year of Omicron | 5 |
| **sTable 4.** Risks for infection in relation to recent exposures: 90-day surveillance periods | 6 |
| **sTable 5.** Risks for infection in relation to remote exposures: 45-day surveillance periods | 7 |
| **sTable 6.** Age-stratified infection risks in relation to recent exposures: 90-day surveillance periods | 8 |
| **sTable 7.** Age-stratified infection risks in relation to remote exposures, 45-day surveillance periods | 9 |
|  |  |
| **sFigure 1.** Study sampling flow diagram | 10 |
| **sFigure 2.** Ascertainment of COVID infection events | 11 |
| **sFigure 3.** Histogram of infection frequency tracked over time | 12 |
| **sFigure 4.** Temporal trends in infection risks over the course of the study period | 13 |
| **sFigure 5.** Temporal trends in SARS-CoV-2 exposures for the source cohort | 15 |

**sTable 1. Characteristics of the study cohort, source cohort, and health system cohort.** Characteristics of the main study cohort of N=4496 longitudinally engaged participants, the source study cohort of N=9224 individuals originally enrolled in the SARS-CoV-2 serological surveillance study, and a random sampling of N=15,000 patients who received outpatient care in our health system during the study period.

|  | **Study Cohort** | **Source Cohort** | **Contemporaneous Health System Cohort** |
| --- | --- | --- | --- |
|  | N=4496 | N=9224 | N=15000 |
| Age in years, mean (SD) | 53.63 (14.78) | 52.81 (15.12) | 54.22 (21.14) |
| Male sex (%) | 1652 (36.7) | 3455 (37.5) | 7164 (47.8) |
| Ethnicity, hispanic (%) | 562 (12.5) | 1492 (16.2) | 3107 (20.7) |
| Race (%) |  |  |  |
| American Indian or Alaska Native | 15 (0.3) | 40 (0.4) | 69 (0.5) |
| Asian | 680 (15.1) | 1180 (12.8) | 785 (5.2) |
| Black or African American | 208 (4.6) | 434 (4.7) | 3668 (24.5) |
| Native Hawaiian or other Pacific Islander | 53 (1.2) | 88 (1.0) | 45 (0.3) |
| White | 2881 (64.1) | 5348 (58.0) | 8977 (59.8) |
| Other | 206 (4.6) | 484 (5.2) | 1338 (8.9) |
| Multiple | 175 (3.9) | 336 (3.6) | 0 (0.0) |
| Unknown | 278 (6.2) | 1314 (14.2) | 118 (0.8) |
| Employee (%) | 1897 (42.2) | 3159 (34.2) | 579 (3.9) |
| Elixhauser comorbidity index, mean (SD) | 4.5 (11.16) | 4.39 (10.89) | 5.67 (12.46) |

**sTable 2. Study cohort characteristics by time periods of eligibility for new infection.**

|  |  | **Surveillance Time Periods** | | | |  |
| --- | --- | --- | --- | --- | --- | --- |
|  | **Overall** | **12/15/21-3/14/22** | **3/15/22-6/14/22** | **6/15/22-9/14/22** | **9/15/22-12/22/22** |  |
| No. observations at risk | 13836 | 4496 | 3796 | 3191 | 2353 |  |
| No. infection events (%) | 2053 (14.8) | 585 (13.0) | 475 (12.5) | 634 (19.9) | 359 (15.3) | <0.001 |
| **Exposure** |  |  |  |  |  |  |
| Vaccine as prior exposure (%) | 13423 (97.0) | 4279 (95.2) | 3696 (97.4) | 3132 (98.2) | 2316 (98.4) | <0.001 |
| Monovalent (%) | 13146 (88.0) | 4279 (100) | 3696 (100) | 3132 (100) | 2039 (88.0) |  |
| Bivalent (%) | 277 (12.0) | 0 (0) | 0 (0) | 0 (0) | 277 (12.0) |  |
| Time since last exposure (%) |  |  |  |  |  | <0.001 |
| 0 to 90 days | 5220 (37.7) | 2880 (64.1) | 792 (20.9) | 1048 (32.8) | 500 (21.2) |  |
| 90 to 180 days | 4558 (32.9) | 873 (19.4) | 2393 (63.0) | 605 (19.0) | 687 (29.2) |  |
| 180 to 270 days | 2416 (17.5) | 354 (7.9) | 468 (12.3) | 1269 (39.8) | 325 (13.8) |  |
| 270 to 360 days | 1316 (9.5) | 378 (8.4) | 90 (2.4) | 165 (5.2) | 683 (29.0) |  |
| ≥360 days | 326 (2.4) | 11 (0.2) | 53 (1.4) | 104 (3.3) | 158 (6.7) |  |
| Exposure category (%) |  |  |  |  |  | <0.001 |
| Vaccine in last 0 to 90 days | 5091 (36.8) | 2751 (61.2) | 792 (20.9) | 1048 (32.8) | 500 (21.2) |  |
| Vaccine in last 90 to 180 days | 4428 (32.0) | 818 (18.2) | 2318 (61.1) | 605 (19.0) | 687 (29.2) |  |
| Vaccine in last 180 to 270 days | 2359 (17.0) | 347 (7.7) | 459 (12.1) | 1228 (38.5) | 325 (13.8) |  |
| Vaccine in last 270 to 360 days | 1267 (9.2) | 363 (8.1) | 87 (2.3) | 158 (5.0) | 659 (28.0) |  |
| Vaccine in last ≥360 days | 278 (2.0) | 0 (0.0) | 40 (1.1) | 93 (2.9) | 145 (6.2) |  |
| Infection in last 0 to 90 days | 129 (0.9) | 129 (2.9) | 0 (0.0) | 0 (0.0) | 0 (0.0) |  |
| Infection in last 90 to 180 days | 130 (0.9) | 55 (1.2) | 75 (2.0) | 0 (0.0) | 0 (0.0) |  |
| Infection in last 180 to 270 days | 57 (0.4) | 7 (0.2) | 9 (0.2) | 41 (1.3) | 0 (0.0) |  |
| Infection in last 270 to 360 days | 49 (0.4) | 15 (0.3) | 3 (0.1) | 7 (0.2) | 24 (1.0) |  |
| Infection in last ≥360 days | 48 (0.3) | 11 (0.2) | 13 (0.3) | 11 (0.3) | 13 (0.6) |  |
| **Demographics** |  |  |  |  |  |  |
| Age, mean years (SD) | 54.6 (14.7) | 53.6 (14.8) | 54.4 (14.7) | 55.1 (14.6) | 56.4 (14.4) | <0.001 |
| Male sex (%) | 5137 (37.1) | 1652 (36.7) | 1412 (37.2) | 1185 (37.1) | 888 (37.7) | 0.88 |
| Hispanic ethnicity (%) | 1663 (12.0) | 562 (12.5) | 441 (11.6) | 387 (12.1) | 273 (11.6) | 0.58 |
| Race (%) |  |  |  |  |  | 0.87 |
| American Indian or Alaska Native | 41 (0.3) | 15 (0.3) | 13 (0.3) | 9 (0.3) | 4 (0.2) |  |
| Asian | 2111 (15.3) | 680 (15.1) | 591 (15.6) | 491 (15.4) | 349 (14.8) |  |
| Black or African American | 639 (4.6) | 208 (4.6) | 165 (4.3) | 149 (4.7) | 117 (5.0) |  |
| Native Hawaiian or other Pacific Islander | 8877 (64.2) | 2881 (64.1) | 2445 (64.4) | 2033 (63.7) | 1518 (64.5) |  |
| White | 158 (1.1) | 53 (1.2) | 48 (1.3) | 39 (1.2) | 18 (0.8) |  |
| Other | 594 (4.3) | 206 (4.6) | 156 (4.1) | 134 (4.2) | 98 (4.2) |  |
| Multiple | 513 (3.7) | 175 (3.9) | 142 (3.7) | 118 (3.7) | 78 (3.3) |  |
| Unknown | 903 (6.5) | 278 (6.2) | 236 (6.2) | 218 (6.8) | 171 (7.3) |  |
| Healthcare employee (%) | 5645 (40.8) | 1897 (42.2) | 1560 (41.1) | 1278 (40.1) | 910 (38.7) | 0.031 |
| **Past Medical History (%)** |  |  |  |  |  |  |
| Hypertension | 3855 (27.9) | 1202 (26.7) | 1037 (27.3) | 910 (28.5) | 706 (30.0) | 0.024 |
| Diabetes | 1373 (9.9) | 426 (9.5) | 362 (9.5) | 326 (10.2) | 259 (11.0) | 0.17 |
| Coronary heart disease or heart failure | 2591 (18.7) | 789 (17.5) | 691 (18.2) | 615 (19.3) | 496 (21.1) | 0.003 |
| Asthma or COPD | 2169 (15.7) | 689 (15.3) | 594 (15.6) | 504 (15.8) | 382 (16.2) | 0.80 |
| Cancer | 2220 (16.0) | 685 (15.2) | 598 (15.8) | 513 (16.1) | 424 (18.0) | 0.026 |
| Autoimmune | 1954 (14.1) | 607 (13.5) | 531 (14.0) | 457 (14.3) | 359 (15.3) | 0.25 |
| Organ transplant recipient | 1800 (13.0) | 548 (12.2) | 476 (12.5) | 426 (13.4) | 350 (14.9) | 0.012 |
| Elixhauser comorbidity index, mean (SD) | 5.1 (11.7) | 4.5 (11.2) | 4.9 (11.5) | 5.3 (11.9) | 6.3 (12.6) | <0.001 |

**sTable 3. Multivariable-adjusted risks for new infection during the first year of Omicron.**

| **Covariates** | **Odds Ratio (95% CI)** | **P value** |
| --- | --- | --- |
| Age decade | 0.87 (0.84, 0.90) | <0.001 |
| Male sex | 1.07 (0.97, 1.19) | 0.18 |
| Hispanic ethnicity | 1.14 (0.99, 1.32) | 0.06 |
| Non-white race | 0.86 (0.77, 0.95) | 0.004 |
| Healthcare employee | 1.00 (0.91, 1.12) | 0.93 |
| Elixhauser comorbidity index | 0.99 (0.99, 1.00) | 0.002 |
| Exposure Period |  |  |
| 12/15/21 | Referent |  |
| 3/15/22 | 0.89 (0.77, 1.03) | 0.11 |
| 6/15/22 | 1.65 (1.43, 1.90) | <0.001 |
| 9/15/22 | 1.23 (1.04, 1.46) | 0.018 |
| No. total prior exposures | 0.91 (0.84, 0.98) | 0.014 |
| Last known exposure |  |  |
| Vaccine: Remote | Referent |  |
| Vaccine: Recent | 0.82 (0.73, 0.93) | 0.002 |
| Infection: Remote | 1.29 (0.94, 1.76) | 0.12 |
| Infection: Recent | 0.14 (0.05, 0.45) | 0.001 |

*Estimates are for odds of new-onset infection within the subsequent 90-day period, where the primary covariates of interest are type and timing of last known exposure. The last known exposure was updated over time to reflect whether the last known exposure was recent within 90 days or remotely occurring 90 days or more previously. All estimates are derived from the multivariable model that adjusted for all the covariates listed in the table and total number of prior exposures in addition to time window (i.e. sequential 90-day time periods starting from December 15, 2021, during which background infections rates and predominant subvariant types tended to shift).

**sTable 4. Multivariable-adjusted risks for new infection during the first year of Omicron in relation to recent exposures: 90-day surveillance periods.**

| **Referent** | **Exposure Group** | **Odds Ratio (95% CI)** | **P value** |
| --- | --- | --- | --- |
| Recent vaccine | Remote vaccine | 1.21 (1.07, 1.37) | **0.002** |
|  | Remote infection | 1.56 (1.13, 2.16) | **0.007** |
|  | Recent infection | 0.17 (0.05, 0.55) | **0.003** |
| Recent infection | Remote vaccine | 7.05 (2.22, 22.45) | **0.001** |
|  | Recent vaccine | 5.81 (1.83, 18.43) | **0.003** |
|  | Remote infection | 9.08 (2.75, 29.94) | **<0.001** |

*Estimates are for odds of new-onset infection within the subsequent 90-day period, where the primary covariates of interest are type and timing of last known exposure. The last known exposure was updated over time to reflect whether the last known exposure was recent within 90 days or remotely occurring 90 days or more previously. All estimates are derived from the multivariable model that adjusted for age, sex, race, ethnicity, healthcare employee status, Elixhauser comorbidity index, and total number of prior exposures in addition to time window (i.e. sequential 90-day time periods starting from December 15, 2021, during which background infections rates and predominant subvariant types tended to shift).

**sTable 5. Multivariable-adjusted risks for new infection in relation to remote exposures during the first year of Omicron: 45-day surveillance periods.**

| **Referent** | **Exposure Group** | **Odds Ratio (95% CI)** | **P value** |
| --- | --- | --- | --- |
| Remote vaccine | Recent vaccine | 0.84 (0.75, 0.95) | **<0.001** |
|  | Remote infection | 1.36 (1.00, 1.84) | **0.05** |
|  | Recent infection | 0.17 (0.05, 0.54) | **<0.001** |
| Remote infection | Remote vaccine | 0.74 (0.54, 1.00) | **0.05** |
|  | Recent vaccine | 0.62 (0.45, 0.85) | **<0.001** |
|  | Recent infection | 0.12 (0.04, 0.41) | **<0.001** |

*Estimates are for odds of new-onset infection within the subsequent 45-day period, where the primary covariates of interest are type and timing of last known exposure. The last known exposure was updated over time to reflect whether the last known exposure was recent within 45 days or remotely occurring 45 days or more previously. All estimates are derived from the multivariable model that adjusted for age, sex, race, ethnicity, healthcare employee status, Elixhauser comorbidity index, and total number of prior exposures in addition to time window (i.e. sequential 45-day time periods starting from December 15, 2021, during which background infections rates and predominant subvariant types tended to shift).

**sTable 6. Age-stratified multivariable-adjusted risks for new infection during the first year of Omicron, in relation to recent exposures: 90-day surveillance periods.**

| **Referent** | **Exposure Group** | **Age <60 years** | | **Age ≥60 years** | |
| --- | --- | --- | --- | --- | --- |
|  |  | **Odds Ratio (95% CI)** | **P value** | **Odds Ratio (95% CI)** | **P value** |
| Recent vaccine | Remote vaccine | 1.31 (1.12, 1.53) | 0.001 | 1.09 (0.88, 1.34) | 0.44 |
|  | Remote infection | 1.35 (0.90, 2.03) | 0.15 | 2.04 (1.19, 3.52) | 0.01 |
|  | Recent infection | 0.16 (0.04, 0.64) | 0.01 | 0.24 (0.03, 1.76) | 0.16 |
| Recent infection | Remote vaccine | 8.44 (2.05, 34.78) | 0.003 | 4.53 (0.62, 33.35) | 0.14 |
|  | Recent vaccine | 6.44 (1.58, 26.37) | 0.01 | 4.17 (0.57, 30.69) | 0.16 |
|  | Remote infection | 8.70 (2.02, 37.44) | 0.004 | 8.53 (1.09, 66.87) | 0.041 |

*Estimates are for odds of new-onset infection within the subsequent 90-day period, where the primary covariates of interest are type and timing of last known exposure. The last known exposure was updated over time to reflect whether the last known exposure was recent within 90 days or remotely occurring 90 days or more previously. All estimates are derived from the multivariable model that adjusted for sex, race, ethnicity, healthcare employee status, Elixhauser comorbidity index, and total number of prior exposures in addition to time window (i.e. sequential 90-day time periods starting from December 15, 2021, during which background infections rates and predominant subvariant types tended to shift).

**sTable 7. Age-stratified multivariable-adjusted risks for new infection in relation to remote exposures during the first year of Omicron: 45-day surveillance periods.**

| **Referent** | **Exposure Group** | **Age <60 years** | | **Age ≥60 years** | |
| --- | --- | --- | --- | --- | --- |
|  |  | **Odds Ratio (95% CI)** | **P value** | **Odds Ratio (95% CI)** | **P value** |
| Remote vaccine | Recent vaccine | 0.80 (0.69, 0.93) | **0.004** | 0.92 (0.76, 1.12) | 0.4 |
|  | Remote infection | 1.09 (0.73, 1.61) | 0.69 | 1.93 (1.19, 3.14) | **0.008** |
|  | Recent infection | 0.14 (0.04, 0.59) | **0.007** | 0.25 (0.03, 1.82) | 0.17 |
| Remote infection | Remote vaccine | 0.92 (0.62, 1.37) | 0.69 | 0.52 (0.32, 0.84) | **0.008** |
|  | Recent vaccine | 0.74 (0.50, 1.11) | 0.14 | 0.48 (0.28, 0.80) | **0.005** |
|  | Recent infection | 0.13 (0.03, 0.56) | **0.006** | 0.13 (0.02, 1) | **0.05** |

*Estimates are for odds of new-onset infection within the subsequent 45-day period, where the primary covariates of interest are type and timing of last known exposure. The last known exposure was updated over time to reflect whether the last known exposure was recent within 45 days or remotely occurring 45 days or more previously. All estimates are derived from the multivariable model that adjusted for sex, race, ethnicity, healthcare employee status, Elixhauser comorbidity index, and total number of prior exposures in addition to time window (i.e. sequential 45-day time periods starting from December 15, 2021, during which background infections rates and predominant subvariant types tended to shift).

**sFigure 1. Study sampling flow diagram.**

44 participants without follow-up survey data after December 1, 2021

4633 participants provided at least 2 blood draws

4557 participants had exposure prior to December 15, 2021

9312 participants enrolled in the source cohort

4496 participants included in analyses

2053 participants with new infection events

17 participants missing key covariates data or age <18 years

**sFigure 2. Ascertainment of COVID infection events.** Each unique COVID infection event was adjudicated based on the presence of a probable COVID infection (defined as any positive PCR test, self-report of infection via serial surveys, serum IgG-N >1.4, or a serum IgG-N increase of 1.4-fold from the most recent prior IgG-N test). We considered an IgG-N index level of ≥1.4 as the threshold for determining a recent prior infection, based on validation for high specificity (i.e., > 99%) by the manufacturer and others.^1^ A repeat COVID infection is considered based on probable COVID infection event occurring after resolution of a prior infection (defined as a most recent serum IgG-N <1.2 or more than 90 days have elapsed since the last probable COVID event). For individuals with prior remote infections, a subsequent new-onset infection (i.e. repeat infection) was identified based on a reduction in IgG-N index to <1.2 preceding another IgG-N level rise by at least 40% and to value of ≥1.4.^2^ Using these criteria, distinct COVID infection events were adjudicated as shown in **Panel A**, with an example application for a hypothetical individual with probable multiple infections shown in **Panel B**.

^1^Chew KL, Tan SS, Saw S*,* et al. Clinical evaluation of serological IgG antibody response on the Abbott Architect for established SARS-CoV-2 infection. *Clin Microbiol Infect* 2020;26:1256 e1259-1256 e1211

^2^Edridge AWD, Kaczorowska J, Hoste ACR*,* et al. Seasonal coronavirus protective immunity is short-lasting. *Nature Medicine* 2020;26:1691-1693

**sFigure 3. Histogram of infection frequency tracked over time.** Vertical lines denoting the 90-day surveillance periods used in the primary analyses and 45-day surveillance periods used in the sensitivity analyses.

| 90-day surveillance periods | 45-day surveillance periods |
| --- | --- |
| 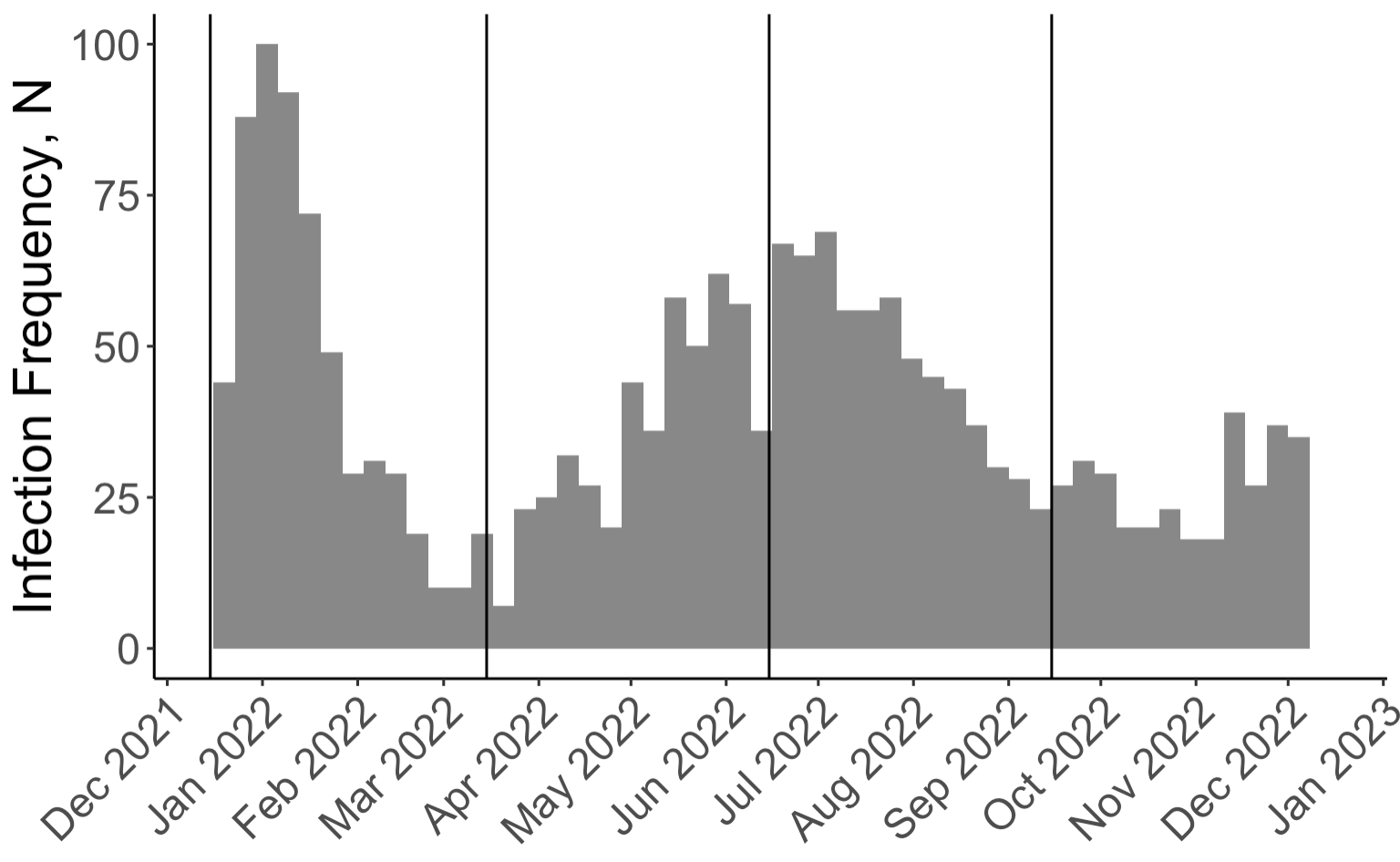 | 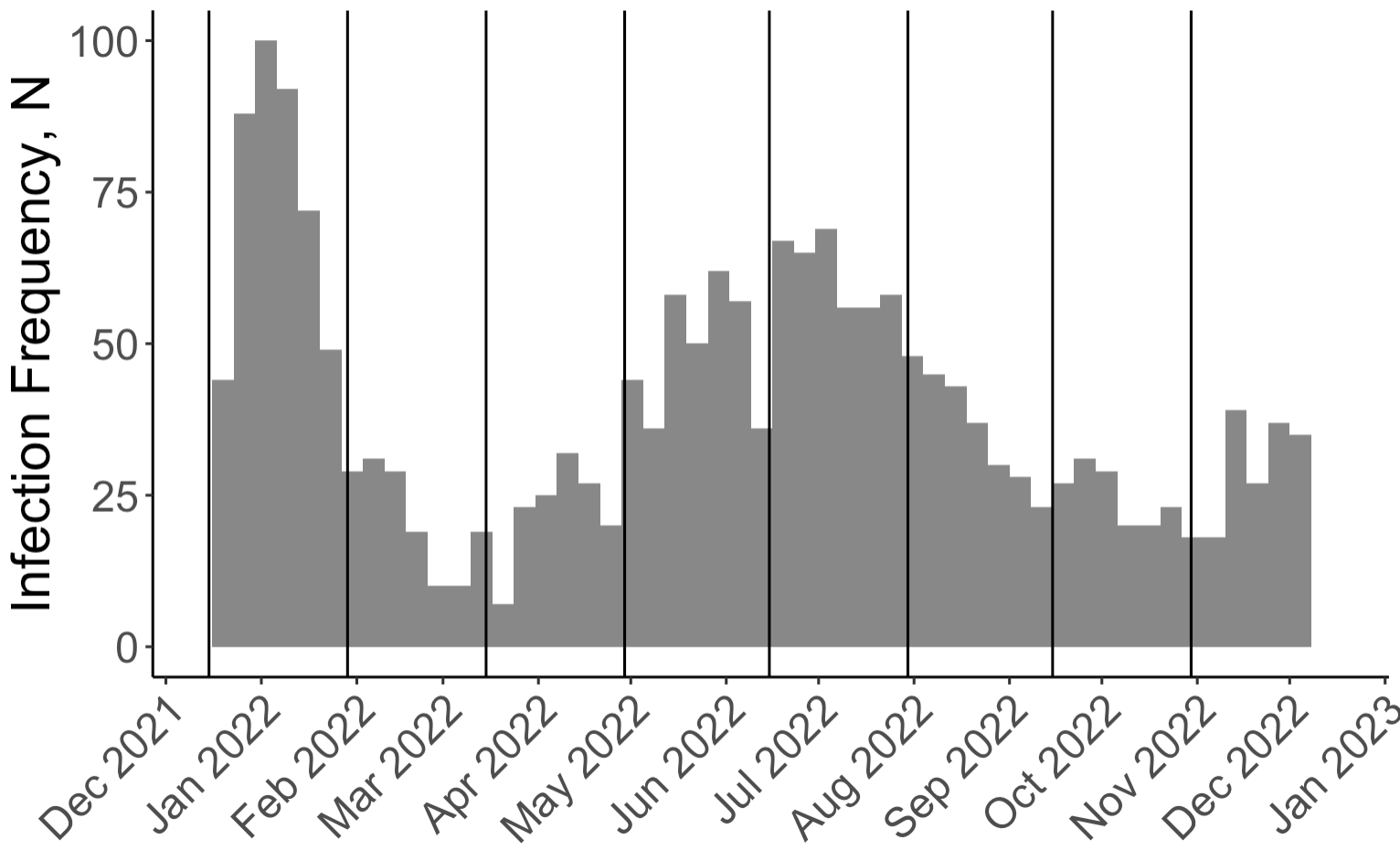 |

**sFigure 4. Temporal trends in infection risks over the course of the study period.** The number of total SARS-CoV-2 tests performed and positive tests documented in our study cohorts is displayed in relation to the total number of tests performed and positive tests catalogued in our regional Los Angeles County area, over time (**Panel A**). The number of unique infections ascertained in our study cohorts is also displayed in relation to the total number of positive tests catalogued in our regional Los Angeles County area, over time (**Panel B**). The regional prevalence of Omicron subvariants during the surveillance period are shown in **Panel C**. Regional data provided by: <http://dashboard.publichealth.lacounty.gov/covid19_surveillance_dashboard/>

**A.**

**B.**

**C.**

**sFigure 5. Temporal trends in SARS-CoV-2 exposures for the source cohort.** Temporal frequency of adjudicated infection and reinfection events as well as vaccinations received are shown for the source cohort.

**A.**

**B.**
